# *Coxiella burnetii *associated reproductive disorders in domestic animals-a critical review

**DOI:** 10.1186/1751-0147-55-13

**Published:** 2013-02-18

**Authors:** Jørgen S Agerholm

**Affiliations:** 1 Section for Veterinary Reproduction and Obstetrics, Department of Large Animal Sciences, Faculty of Health and Medical Sciences, University of Copenhagen, Dyrlægevej 68, DK-1870, Frederiksberg C, Denmark

**Keywords:** *Coxiella burnetii*, Q fever, Reproduction, Abortion, Cattle, Sheep, Goat, Buffalo, Pig, Dog, Cat

## Abstract

The bacterium *Coxiella burnetii* has been detected in the fetal membranes, birth fluids and vaginal mucus, as well as in the milk and other excretions of several domestic mammals. The finding of *C. burnetii* in association with abortion, parturition and in the postpartum period has led to the hypothesis that *C. burnetii* causes a range of reproductive diseases. This review critically evaluates the scientific basis for this hypothesis in domestic mammals.

The review demonstrates a solid evidence for the association between *C. burnetii* infection and sporadic cases of abortion, premature delivery, stillbirth and weak offspring in cattle, sheep and goats. *C. burnetii* induced in-herd epidemics of this complete expression of reproductive failure have been reported for sheep and goats, but not for cattle. The single entities occur only as part of the complex and not as single events such as generally increased stillbirth rate. Studies show that *C. burnetii* initially infects the placenta and that subsequent spread to the fetus may occur either haematogenous or by the amniotic-oral route. The consequences for the equine, porcine, canine and feline conceptus remains to the elucidated but that infection of the conceptus may occur is documented for most species. There is no solid evidence to support a hypothesis of *C. burnetii* causing disorders such as subfertility, endometritis/metritis, or retained fetal membranes in any kind of domestic animal species.

There is a strong need to validate non-pathology based methods such as polymerase chain reaction for their use in diagnostic and research in relation to establishing *C. burnetii* as the cause of abortion and to adapt an appropriate study design and include adequate control animals when linking epidemiological findings to *C. burnetii* or when evaluating effects of vaccination in production herds.

## Introduction

*Coxiella burnetii* is a zoonotic obligate intracellular bacterium that has an almost worldwide distribution. The bacterium has a reservoir in many wild and domestic mammals, birds and arthropods such as ticks. The infection causes Q fever in humans. Infection with *C. burnetii* in man is usually asymptomatic or resembles a flu-like infection although more severe conditions such as endocarditis, pneumonia and hepatitis may develop [1].

The term Q fever has been adapted in veterinary medicine although “Q fever” (query fever) refers to a febrile illness originally observed in abattoir workers in Australia [2] and despite another clinical course in animals than in man. This terminology has been maintained although coxiellosis may be a more appropriate term, especially in cases without fever.

Infection with *C. burnetii* occurs worldwide in domestic ruminants as indicated by presence of seropositive animals as recently reviewed by Guatteo *et al.*[3]. Despite this, knowledge on acute infection is almost absent. Culturing demands growth in embryonated eggs or cell cultures and requires biosafety level 3 facilities. Similar facilities are needed for experimental infections. Access to such facilities is usually limited and studies on large animals are costly and often impractical due to facility limitations. Furthermore, investigation of spontaneous Q fever infections in domestic animals was until recently hampered by the lack of cheap, sensitive and specific laboratory methods such as polymerase chain reaction (PCR) and enzyme-linked immunosorbent assay (ELISA). However, it is generally accepted that chronic infection with *C. burnetii* may cause abortion, premature birth, dead or weak offspring in cattle, sheep and goats [4-6] but other reproductive conditions in cattle have also been claimed to be associated with *C. burnetii*. However, in depth reviews focusing on the known implications of Q fever on reproduction in each species are lacking. There are biological indications of species differences in relation to the impact on reproduction and recent molecular studies have shown that different strains of *C. burnetii* exist and that strains are associated with different ruminant hosts although cross infection does occur [7-10].

Recently commercial vaccines have become available for immunisation of ruminants. These may be used to reduce the zoonotic risks of Q fever in domestic ruminants and they have been used to reduce excretion of *C. burnetii* from goats in recent Q fever outbreaks in the Netherlands ex. [11-16], but they are also marketed to prevent or reduce some of the reproductive aspects of ruminant Q fever that have been claimed to exist such as metritis, retained fetal membranes, infertility, sterility, mastitis and increased herd prevalence of abortion and stillbirth. There is an obvious need to critically review the literature before vaccination is recommended to prevent reproductive problems and scientifically evaluate if Q fever is causally associated with reproductive diseases in general. The aim of this review is therefore to critically review reported associations between *C. burnetii* and reproduction in domestic mammals.

### General considerations

The search strategy and selection criteria for references are provided as [Additional file 1].

Before dealing with Q fever in detail, one need to understand the general pathogenesis of placental and fetal infection applied to a wide range of pathogens. This background knowledge is needed to understand the intrauterine dynamics of *C. burnetii* infections and to interpret laboratory findings in cases of reproductive failure associated with *C. burnetii*. Furthermore, a few remarks are given on definitions as case definitions are lacking in many studies.

#### Abortion, premature delivery, stillbirth and weak offspring (APSW) complex

**Figure 1:**
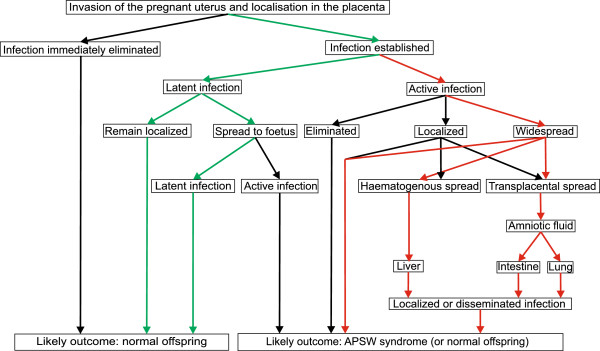
**Schematic outcomes of an intrauterine infection with *Coxiella burnetii *in a pregnant animal. **Little knowledge on the intrauterine spread of *C. burnetii *is present, but data indicates that the infection may follow one of two routes after an initial localization in the placenta (indicated by red and greens arrows). A latent infection (green arrows) that either remains localized in the placenta or spreads to the fetus (still latent) is probably the most common outcome, at least in cattle. This situation is characterised by normal offspring that may or may not be congenitally infected and vaginal excretion of organisms in association with parturition and in the postpartum period. An active infection (red arrows) that may remain limited to the placenta, although being widespread, or may spread to the fetus by the haematogenous or the amniotic-oral route will most likely compromise the fetus and cause abortion, premature delivery, stillbirth and weak offspring (APSW Complex) although normal but probably congenitally infected offspring may also be found.

The outcome of an infection of the pregnant uterus can be a range of conditions, including abortion, delivery of premature offspring, stillbirth and weak offspring (here termed APSW Complex) in addition to clinically normal progeny that may or may not be congenitally infected. The complexity of the events that may lead to these different outcomes is illustrated in Figure 1. It is imperative to understand this complexity and the different ways an infection may develop in the placenta and fetus when interpreting laboratory data of diseased offspring. It is also important to recognise that conditions such as stillbirth and weak offspring cannot be regarded as isolated conditions but as possible outcomes of an intrauterine infection embracing the entire APSW Complex. The outcome of an intrauterine infection with *C. burnetii* depends on (but not limited to) strain virulence, maternal and fetal immune responses, severity of placental infection/lesion, possible spread to and dissemination in the fetus, gestation age, and number of infected fetuses. Adapted to the field situation, this means that in-herd epidemic Q fever should only be suspected if the entire APSW Complex occurs, but not if only one condition such as increased stillbirth rate occurs.

#### Infertility, subfertility and sterility

Infertility, subfertility and sterility are used interchangeably in papers on Q fever and usually without stating the basis for the diagnosis. Infertility and subfertility are synonyms and refer to a diminished capacity to produce offspring while sterility means a complete (absolute) inability to produce offspring [17]. These terms cover a very heterogeneous group of disorders and extensive examinations are usually needed to establish such a diagnosis. In this review subfertility and sterility is only used if the conditions occur as independent conditions or as complications to Q fever, but when referring to original studies the authors’ use is maintained although being imprecise and without knowledge of the basis for the diagnosis. My use of these terms is avoided in situations where they are secondary and misleading, e.g. an animal that has even a single abortion is per definition subfertile although she may produce normal offspring in the future.

#### Endometritis and metritis

Endometritis and metritis refer to superficial (endometrial) and profound inflammation of uterus, respectively and their strict use requires histopathological examination. In clinical research, inflammation of the postpartum uterus is divided into puerperal metritis, clinical endometritis, subclinical endometritis and pyometra [18]. With a few exceptions, case definitions have not been provided in published studies.

#### Retained fetal membranes

Retention of the fetal membranes is a common condition in dairy cattle. The fetal membranes are considered retained if they are not expulsed within 24 h postpartum [19]. Case definitions have not been included in studies on associations between *C. burnetii* and retained fetal membranes, so some authors may have used other definitions.

### Cattle

Studies done in cattle before strict biosafety measurements were implemented have shown that seronegative cows develop a transient fever 2–3 days after subcutaneous (sc) inoculation with *C. burnetii* Nile Mile strain (tick origin) at a dose of 4×10^8^ guinea pig doses. Of two non-vaccinated controls, one cow delivered a full-term stillborn calf with apparent *C. burnetii* dissemination 178 days after inoculation. The other cow aborted after 149 days of unknown cause as the fetus was lost [20]. Acute infection was also studied by Plommet *et al.*[21] who inoculated twelve 8 to 11-month-old non-pregnant heifers by *C. burnetii* strain C9 by the intradermal route. The heifers developed a febrile response of 40–41°C within 24–36 h associated with an acute self-curing pneumonia. The body temperature decreased to normal level within 1 week. The heifers were inseminated at the age of 16 months with various results, but evidence is not provided that the poor outcome of insemination was due to *C. burnetii* as a wide range of other possible causes exists. There is no experimental evidence to support that *C. burnetii* causes abortion in cattle as the only reliable case was a full-term stillborn calf [20].

**Figure 2:**
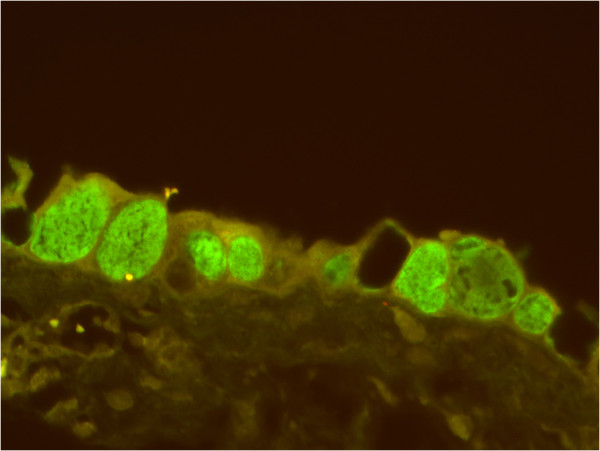
**Trophoblasts infected by *Coxiella burnetii*. **Huge amounts of *C. burnetii *DNA are seen as green fluorescence within distended trophoblasts. Fluorescence in situ hybridization, placenta, goat. Courtesy of TK Jensen, Danish Veterinary Institute, Technical University of Denmark.

Determination of the abortifacient potential of *C. burnetii* is complicated as this organism is commonly detected in the placenta, birth products and vaginal mucus after abortions as well as after normal parturition [22-28]. Confirmation of an association between lesions and presence of the organism is therefore mandatory to confirm *C. burnetii* as the cause of fetal disease – a demand generally applied in diagnostic reproductive pathology. Examination of spontaneous bovine abortion cases submitted to diagnostic laboratories has demonstrated that *C. burnetii* is associated with placentitis and probably subsequent abortion in cattle by fulfilling this criterion [29]. Gross lesions vary from insignificant to haemorrhagic and necrotising placentitis, while the fetus usually seems unaffected, although autolytic. Similar, microscopic lesions range from severe extensive inflammation dominated by necrosis, haemorrhage, vasculitis, oedema and large numbers of neutrophils to mild inflammation with scattered foci of necrotic trophoblasts and sparse infiltration with mononuclear cells. In representative cases, trophoblasts are distended due to cytoplasmic accumulation of huge numbers of fine, basophilic stained organisms [29-33]. While severe inflammation generally is accepted to induce abortion, the interpretation of infection associated with sparse or no lesions is speculative. A confirmatory diagnosis and better visualisation of bacteria can be obtained by immunohistochemistry (IHC) [29,31,33] or fluorescence in situ hybridization (FISH) [32] (Figure 2), although older studies have used histochemical staining methods such as Macchiavello, Stamp and Köster stains [30,34].

Although the infection may remain confined to the placenta, spread of the infection to the fetus may occur by the amniotic-oral route, if bacteria penetrate the placenta, contaminate the amniotic fluid and become aspirated/swallowed by the fetus (Figure 1). In such cases, bacteria become established in the intestinal tract and may invade the lungs by the trachea-bronchial route thus inducing bronchopneumonia. In fact, Bildfell *et al.*[29] found bronchopneumonia in 2 out of 6 cases and Cantas *et al.*[35] found bacterial DNA by PCR in the stomachs of 18 out of 51 bovine abortions. However, haematogenous spread to the fetus, probably through the umbilical vessels as seen in some bacterial infections may also occur as indicated by the finding of bacteria in multiple tissues in a stillborn calf [20].

Q fever abortion is often diagnosed in late term fetuses; however this may reflect that late term fetuses are submitted for examination more often than less developed fetuses [29,33,36]. However, prevalence of antibodies against *C. burnetii* is more frequent in cows that have aborted (due to undetermined cause) in the last trimester than in first and second trimester cows [37], but the significance of this is unknown. Knowledge on the capacity of *C. burnetii* to infect and damage the conceptus during the entire gestation period is lacking, but the placenta is often infected at some time during gestation without apparent effect on the fetus [22,23,28]. Such an event may induce a maternal antibody response and explain the apparent higher prevalence of seropositive cows with increasing gestation age.

*C. burnetii* seems to act as a primary pathogen although co-infection with other organisms obviously occurs by chance. Seasonal variation in abortion risk has not been registered [29,33], but the prevalence of seropositive cows seems to be highest in the autumn [37].

*C. burnetii* infection has been reported in just a few stillborn calves [29,31,33]. These probably represent sporadic fetal infections with a fetus surviving to the end of the gestation period and it is most likely that the entire spectrum of the APSW complex would be identified if sufficient numbers of calves were examined. The herd rate of perinatal mortality, including stillbirth, was not associated with the level of antibodies against *C. burnetii* in bulk tank milk [38]. There is no evidence suggesting that *C. burnetii* per se should be a significant cause of stillbirth or weak neonatal calves.

*C. burnetii* associated abortion in cattle is usually not diagnosed even in larger surveys on causes of abortion in regions were the infection in endemic [39,40] and studies focused on Q fever and abortion concurrently conclude that *C. burnetii* is an infrequent cause of abortion in cattle [29,30,32,33]. The abortion rate associated with *C. burnetii* corresponds to that of opportunistic pathogenic bacteria such as staphylococci and streptococci but lower than e.g. *Trueperella pyogenes* and fungi [32,39,41]. There is no evidence for *C. burnetii* being associated with herd outbreaks of abortion in cattle.

A number of studies have used PCR to evaluate the possible role of *C. burnetii* in bovine abortion. Parisi *et al.*[24] and Clemente *et al.*[27] found 17.2% and 11.6% PCR positive animals among cattle that had aborted, respectively. Real time PCR has been claimed to be a reliable tool in diagnosing Q fever abortion. However, assessment of this method against the gold standard in diagnostic reproductive pathology, agent identification with corresponding lesions, has not been published and the method must at present be regarded unreliable to identify the cause of abortion, especially because of the frequent placental infection in apparently healthy cows [22,23,28]. Vaginal excretion of *C. burnetii* is usually <14 days in cows that abort due to an unknown cause but excrete *C. burnetii* at the day of abortion [42]. However, PCR methods are not reliable techniques to determine the cause of abortion as stated previously and the prevalence found in groups of animals that have experienced abortion merely reflects the combined frequency of “true” *C. burnetii* associated abortion cases and animals just having vaginal *C. burnetii* excretion in the postpartum period. The latter constitutes the vast majority of positives and reflects the population infection prevalence.

Detection of antibodies against *C. burnetii* in maternal blood samples in cases of abortion has been done in a number of studies to investigate if seropositive cows abort more frequent than seronegatives [37,43-45]. An epidemiological study based on 287 cases of abortion and 1318 age matching controls demonstrated a similar prevalence of seropositive animals thus strongly suggesting that the abortion risk is not influenced by presence of maternal antibodies [45]. Ruiz-Fons *et al.*[46] did not find a significant difference in prevalence of *C. burnetii* antibodies in beef cattle herds with a recent history of abortion and those without. Other studies have indicated an increased risk in seropositive animals [37,43,47]. It is however, imperative to recognise that *Neospora caninum* associated abortions are more likely to occur in herds with antibodies to *C. burnetii* than in seronegative herds [48]. It is very likely that an increased abortion rate is due to *N. caninum* rather than *C. burnetii* as *N. caninum* is a major abortifacient in cattle [49]. It also emphasises the need for thorough diagnostic examinations when studying the abortifacient potential of *C. burnetii*.

Examination for fetal antibodies is used in abortion diagnostic for certain pathogens in immunocompetent fetuses e.g. [50,51]. Presence of antibodies may indicate infection of the conceptus and would be valuable knowledge when investigating the effects by *C. burnetii* on the fetus. Fetal IgM antibodies against *C. burnetii* have been demonstrated after an experimental maternal infection [20]. This indicates that the fetus can develop a humoral immune response to *C. burnetii*.

A number of studies have addressed possible associations between *C. burnetii* (i.e. excretion or/and antibodies) and a range of more or less well-defined reproductive conditions such as retained fetal membranes [43,52-54], conception rates and calving outcome [44,47,53-56], infertility and sterility [52,55,57,58], and endometritis/metritis [53-56,58,59]. The studies show that *C. burnetii* can be detected in some cases, which is not surprising knowing that *C. burnetii* is excreted by healthy cows by different routes including vaginal [22-28] and obviously also by some diseased cattle simply by coincidence. Similar, some diseased cattle are seropositive by chance because of the widespread occurrence of the infection [3]. However, evidence of an association between *C. burnetii* infection and any of the conditions mentioned has not been provided. Some of the studies unfortunately miss adequate clinical and epidemiological elements such as appropriate controls, clear case definitions, and statistical evaluation – lacks that may lead to overestimation of the significance of *C. burnetii* excretion or presence of antibodies. The importance of an appropriate study design that includes adequate control animals cannot be overemphasized when dealing with an infection that is present in many healthy animals. This also refers to vaccination studies where the influence of *C. burnetii* is evaluated indirectly as farmers may cull “problem-animals” and change awareness on parameters that are measured and thereby obviously induce a positive effect on herd reproduction. Also, reproduction parameters fluctuates over time and changes may coincidence with vaccination and be misinterpreted as a vaccination effect unless proper controls have been included.

In conclusion, evidence has not been provided that shows causation between *C. burnetii* and poor conception rates, subfertility/infertility, sterility, retained placenta, or endometritis/metritis neither at individual level nor at herd level. In fact, a recent study [54] showed that seropositive shedding cows had better reproduction than non-infected cows. Consequently there is at present no scientific basis for preventing these conditions by vaccination against Q fever. The association between *C. burnetii* and aspects of reproduction in cattle and other domestic animals are summarized in Table 1.

**Table 1:** **Summary of scientific evidence for *Coxiella burnetii *associated reproductive disorders in domestic mammals**

	**APSW complex**^**1**^		**Endometritis/metritis**	**Subfertilty**^**2**^**/sterility**	**Mastitis**		**Retained placenta**
	**Sporadic**	**Epidemic**^**3**^			**Subclinical**	**Clinical**	
Cattle	+	−	−	−	(+)^4^	−	−
Sheep	+	+	−	−	−	−	−
Goat	+	+	−	−	−	−	+^5^
Buffalo	−^6^	−	−	−	−	−	−
Horse	−	−	−	−	−	−	−
Pig	−	−	−	−	−	−	−
Dog	−	−	−	−	−	−	−
Cat	−^7^	−	−	−	−	−	−

^1 ^Abortion, Premature delivery, Stillbirth and Weak offspring Complex.

^2 ^ Other than due to single event abortion.

^3 ^Herd level.

^4 ^Based on one study in one herd [62].

^5 ^Based on one study in one herd [91] and just as a complication to the APSW complex.

^6 ^Placental/fetal infection has been reported [98].

^7 ^Q fever in man has been associated with cats having stillborn kittens although the kittens were not examined.

It is well established that *C. burnetii* is excreted in milk ex. [25,26,60] and it has been isolated from udder tissue and corresponding lymph nodes [57,61] and therefore obviously also from cases of mastitis [43,58]. A single well-conducted study in a single herd has indicated an association between subclinical mastitis and *C. burnetii*[62].

Knowledge on Q fever in relation to the reproduction of bulls is almost lacking. A single study demonstrates that *C. burnetii* may be present in semen and venereal transmission of the infection is thus possible [63]. The role of such transmission for female reproduction remains to be elucidated.

### Sheep

Acute infection has been studied in pregnant ewes inoculated by the intravenous (iv) or intraperitoneal routes with the ovine *C. burnetii* strain Tchilnov. The ewes developed fever up to 40.9°C for 2–3 days 5–7 days after exposure followed by reappearance of a slight fever on post inoculation days 12–13. Fever was accompanied by depression, salivation, rhinitis, conjunctivitis and tachypnea (interstitial pneumonia). Several days before lambing, the ewes’ general condition deteriorated and they lambed with full-term stillborn or weak non-viable lambs accompanied by a necrotic and inflamed placenta. The bacterium was found in the placenta [64]. Six ewes, pregnant around day 100, were inoculated sc by the *C. burnetii* Nine Mile strain in another study [65]. Acute clinical signs were not reported but the ewes lambed with noticeably small and weak lambs. A necrotic placenta accompanied one lamb that died 2-days-old. *C. burnetii* was isolated from the placenta in 5 out of 6 ewes and in 2 out of 2 amnion fluid samples. The acute clinical course in spontaneous cases has not been reported. Berri *et al.*[66,67] did not report symptoms in laboratory flocks accidentally exposed to *C. burnetii* thus indicating that clinical signs may be unapparent.

Determination of the abortifacient potential of *C. burnetii* for sheep is complicated for the same reasons as for cattle, i.e. excretion of bacteria from apparently healthy animals [26,66,68-73] and therefore confirmatory histopathology is needed in addition to agent detection to determine *C. burnetii* as the cause of abortion.

Examination of spontaneous cases of late-term abortions has demonstrated mucopurulent exudates and focal to coalescing necrosis of cotyledons and intercotyledonary placenta. In some cases, intercotyledonary lesions may be prominent. Some fetuses seem to be of reduced body condition. Histologically, a severe necrotising placentitis accompanied by prominent neutrophilic infiltration, oedema and haemorrhage in the stroma and widespread cytoplasmic accumulation of basophilic and IHC or FISH positive bacteria in trophoblasts are seen. Foci of hepatic necrosis or granulomatous inflammation have been found in some cases, but association with *C. burnetii* remains to be determined. PCR analysis of multiple tissues from aborted lambs with PCR positive placenta, but without a histopathologically confirmed cause of abortion, have revealed *C. burnetii* DNA in multiple tissues. [27,30,31,35,64,74-80]. These findings indicate that *C. burnetii* may infect the fetus itself by the aminotic-oral route and haematogenously (Figure 1). The full spectrum of the APSW complex has been demonstrated. Aborted fetuses have been late term abortions but it is unknown if *C. burnetii* causes fetal losses throughout the entire gestation period.

Several studies mention that *C. burnetii* causes epidemic herd outbreaks of the ASPW complex. Zeman *et al.*[76] mention that the rate of late term abortions and weak lambs varied from 10 to 60% within groups in a flock of sheep through a 3-month-period, while around 23% aborted in a flock of Austrian dairy sheep [77]. Rády *et al.*[30] mention that “large numbers of abortions occurred within a short period”. However, *C. burnetii* is not always associated with epidemic abortion as Marmion and Watson [81] reported only 3 *C. burnetii* associated abortions in a flock of 101 breeding ewes. In a diagnostic survey Oporto *et al.*[80] found *C. burnetii* infection with corresponding placental lesions in samples from 2 out of 148 farms (1.4%), but not all submitted materials allowed reliable diagnostic, so the prevalence may have been higher. Kirkbride [82] identified *C. burnetii* as the cause of abortion in only 0.1% of the cases in a diagnostic survey of 1,784 abortions and stillbirths in the USA, while a study of 86 ovine abortions in Switzerland revealed a prevalence of 1% [83]. In four individual groups of ewes experiencing abortion, abortion rate from 1.8 to 13.0% was observed. However, toxoplasmosis was found in this herd as well and the diagnosis was based only on PCR, so *C. burnetii* may have been misdiagnosed as cause of abortion as PCR is not a reliable method to diagnose the cause of abortion. The finding is further complicated by an unknown influence of systematic treatment with antibiotics in late gestation [66,67]. PCR has been used as a diagnostic tool in other studies [24,27,35], but interpretation in relation to abortion cause remains blurred. *C. burnetii* is most likely able to cause sporadic as well as epidemic abortion in ewes. Infection with *C. burnetii* during a pregnancy does not influence the outcome of the next pregnancies [71].

Seroepidemiological studies have been performed to investigate association between flock seroprevalence and a recent history of abortion with different outcomes. Ruiz-Fons *et al.*[46] did not find any associations between seroprevalence and previous abortions, while Garcia-Pérez *et al.*[84] found a significant higher seroprevalence in flocks with abortions than in flocks without. However, interpretation remains uncertain as the cause of abortion was not known in any of the studies and as unrecognized associations with other infections may exist as for cattle.

*C. burnetii* has not been associated with other reproductive disorders in sheep than the APSW complex. *C. burnetii* DNA was isolated from different sample types from a pooled group of sheep displaying abortion, repeat breeding, retained fetal membranes, and endometritis. *C. burnetii* was found in some animals but the study does not allow any conclusions regarding possible causations [53]. *C. burnetii* is excreted in the milk ex. [26], but reports on possible associations with subclinical or clinical mastitis in sheep have not been published.

### Goats

Acute infection has been studied in pregnant goats after sc inoculation with the ovine *C. burnetii* strain CbC1 [11,85,86]. A dose depend rise in temperature was observed. Goats given 10^8^ mouse infective doses developed fever to around 40.5°C, while only some goats given 10^6^ doses did so and goats inoculated with 10^4^ doses continuously had rectal temperature below 39.5°C (normal level). The temperature rise started at post inoculation day 3 and lasted for 3 to 5 days. Inoculation was done on either gestation day 84 [11] or 90 [85,86]. Dose independent abortions started to occur on day 25 after infection and throughout the remaining gestation period. Seventy-five per cent of goats given a dose of 10^4^ mouse infective doses on gestation day 84 aborted before gestation day 148 (normal gestation period 150 ± 1.8 days) [11,85,86].

The pathology of experimental *C. burnetii* infections in pregnant goats was studied by Sanchez *et al.*[86]. Goats (n = 12, 90 days pregnant) were inoculated sc with 10^4^ mouse infective doses. Fetuses were either examined when goats were euthanized at gestation day 116 or 130 or when aborted (day 132 ± 4). There was apparently a delay in the development of placental lesions after bacterial invasion of the placenta as *C. burnetii* had infected the intercotyledonary allantochorion and some placentomes on post inoculation day 26 but histopathological changes were either absent or mild. On post inoculation day 40, a widespread severe necrotising and suppurative inflammation had developed in the cotyledons and the intercotyledonary placenta. *C. burnetii* antigen was detected in dilated trophoblasts and free in debris by IHC and confirmed by PCR. Fetuses aborted on post inoculation day 42 ± 4 showed similar lesions. PCR analyses for *C. burnetii* DNA showed that bacterial DNA was present in fetal liver and spleen on post inoculation day 26 and also in the lung, abomasal content and peritoneal fluid on post inoculation day 40 and in abortion cases. The presence of bacteria DNA was usually not accompanied by lesions or positive IHC staining although mild to moderate perivascular hepatitis may be seen [11,85,86]. These findings indicate that fetuses may develop a *C. burnetii* bacteraemia shortly after the colonization of the placenta, at least in experimental settings (Figure 1).

The placental gross morphology and histopathology of spontaneous cases of *C. burnetii* associated abortion in goats resemble the lesions observed in sheep and those found in experimental caprine cases. Significant lesions are often present in the intercotyledonary placenta and macroscopically, the cotyledonary lesions may be less conspicuous. Significant gross or microscopic fetal lesions have not been reported although foci of granulomatous hepatitis have been found as in sheep. Organisms have been observed in several tissues by direct fluorescent antibody test [74,87-89]. The findings in experimental and spontaneous cases indicate that *C. burnetii* associated abortion in goats is mainly due to placental lesions and although bacteriaemia develops, this condition is not associated with detectable lesions in the fetus. The infection may lead to entire spectrum of the APSW complex.

It is difficult to assess the importance of the *C. burnetii* associated APWS complex in goats. In a diagnostic survey based on 211 cases of abortions and stillbirths submitted to diagnostic examination in California, USA, *C. burnetii* was determined as the cause in 19% and in a diagnostic survey performed in Switzerland, *C. burnetii* was identified as the cause of abortion in 10% of 144 abortions [83,90]; figures that are far higher than found in cattle and sheep (around 1% or less) [32,33,39-41,82]. However, comparing diagnostic surveys may be severely biased so direct comparison is not possible. Reports on the prevalence of the APWS complex in goat flocks undergoing an epidemic have indicated a prevalence of 31–93% [74,87-89,91]. There is no reason to believe that *C. burnetii* should not cause sporadic abortion as well but such cases are probably just less frequently published than outbreaks. For the same reasons as mentioned earlier, infections have mostly been reported in late term or full term kids.

The minimum incubation period, i.e. until first abortion occurs, following sc inoculation on gestation day 84 was found to be 39 days [11] and 25 and 38 days in two studies inoculating the CbC1 strain on gestation day 90. The maximal incubation period in the same studies varied from 39 day if inoculated on gestation day 84 to 46–48 days when exposed on gestation day 90 [85,86]. In a case report based on a point source exposure of several goat flocks, the minimum incubation period was 21, 53, and 67 days in three flocks, respectively [89]. A reliable maximum period cannot be established due to possible in-flock circulation of the pathogen after the first abortion.

There is no evidence indicating that *C. burnetii* can induce endometritis per se although placental *C. burnetii* infection and the associated inflammation in case of abortion may cause endometrial inflammation. This inflammation regresses after abortion without treatment [86], probably as part of the postpartum uterine involution. Abortion is usually without premonitory signs and occurs uneventful although dystocia may develop due to fetal death and malposition or uterine inertia [89] although anorexia, depression, agalactia and retained fetal membranes may be seen rarely [91].

A number of studies have focused the prevalence of caprine abortions and other disorders due to *C. burnetii* using PCR or serology [24,27,35,53,79,92,93] with the same limitations as for cattle and sheep as healthy goats may excrete the bacterium in e.g. vaginal mucus [15,26,94,95] and as bacteria may be present in the genital tract of normal does [96]. Although the level of infection as determined by e.g. real time PCR may be positively associated with the risk of abortion [97] and placental inflammation, the diagnostic significance of this has not been proved and further, the concomitant presence of other abortifacients such as *Campylobacter* sp. cannot be excluded.

### Buffalo

A study of 164 aborted Italian water buffalo (*Bubalus bubalis*) fetuses showed an infection prevalence of 8.5% by PCR. The highest infection rate was found in placenta (53.4%) followed by liver (33.3%) and spleen (13.3%) [98]. Excretion by different routes such as vaginal has been reported [53]. The association with APSW complex remains to be elucidated, but it is likely that sporadic cases occur if the infection mimics that in cattle.

### Horse

A serological study in Atlantic Canada has shown that horses may develop specific antibodies following exposure as 13 out of 123 horses were seropositive [99]. In a retrospective study on 407 equine cases of abortion, stillbirths and neonatal death in France, *C. burnetii* DNA was found in lung and placenta of six cases by real time PCR. However, the significance of these findings remains obscure as specific lesions were not found and common equine abortifacients were detected in five out of the six cases [100]. Similar Runge *et al.*[101] detected *C. burnetii* DNA by real time PCR in one out of 23 aborted equine fetuses. This fetus had a concomitant infection with equine herpesvirus type 1. The role of *C. burnetii* in the equine APSW complex remains to be established.

### Pig

Knowledge on porcine Q fever is almost absent. Studies performed in the 1950s have demonstrated the presence of serum antibodies as referenced in [102] and a recent study on feral pigs in Australia showed a seroprevalence of 22.0% [103]. Placentas from 101 sows mostly held on farms with dairy cattle in the United Kingdom were negative by guinea pig inoculation [102]. Stoker in [102] refers to an unpublished study, where a pregnant sow was inoculated. She seroconverted but *C. burnetii* was not excreted in the placenta. However, taking the wide spectrum of hosts for *C. burnetii* into consideration, it seems unlikely that pigs cannot become infected and maybe shed the organism. It remains to be documented if *C. burnetii* causes the APSW complex under certain conditions, but it is possible based on comparative aspects.

### Dog

There is no direct evidence that dogs may develop a reproductive disorder after exposure to *C. burnetii*. However, it is well known that dogs may become infected and develop a humoral immune response e.g. [104], but the clinical aspects remain obscure. But a human outbreak of Q fever has been linked to close contact to a parturient dog that gave birth to three pups that died shortly after birth while a fourth pup died within 24 h. The pups were not examined [105].

### Cat

Viable *C. burnetii* bacteria can be detected in the genital tract of both healthy and diseased cats. The bacterium has been isolated from the vagina of cats having abortion and fever, although this may be an accidental finding [106,107].

Parturient cats delivering stillborn or healthy kittens have been implicated in several outbreaks of Q fever in man. Some cats have had vaginal discharge prior to parturition [108-112]. None of the kittens have been examined as the association between the cat’s parturition and outbreak of Q fever was established in retrospective epidemiological investigations and it remains unknown if *C. burnetii* is associated with the APSW complex in cats.

## Conclusions

Acute infection with *C. burnetii* in domestic animals is usually referred to as subclinical. However, experimental infections have shown that animals may develop a self-curing febrile condition in the first days after exposure. Although direct inoculation of the bacterium at a high dose poorly resembles spontaneous exposure, it seems likely that at least some animals will become febrile. This may remain unnoticed by the breeder or not linked to Q fever. However, Q fever should probably be borne in mind when veterinarians encounter fever of unknown cause in animals and preferable paired serum samples should be taken and analysed.

There is a strong need to validate PCR as a method to determine *C. burnetii* as cause of abortion. Several studies have used PCR for this purpose, but knowing that *C. burnetii* is excreted in the fetal membranes, birth fluids and vaginal mucus, this method is unreliable and PCR most likely overestimates the importance of *C. burnetii* as an abortifacient significantly. It is also important to realize the apparent correlation between *N. caninum* and *C. burnetii* infections in cattle and to exclude other pathogens when examining aborted fetuses for *C. burnetii* irrespectively of species. Detection of *C. burnetii* in association with corresponding lesions is still the gold standard when investigation the possible role of *C. burnetii* in cases of the APSW complex. The association between *C. burnetii* and sporadic cases of the ruminant APSW complex is well established although larger case series are needed to increase the knowledge on the fetal pathogenesis and pathology. *C. burnetii* associated in-herd epidemics of the APSW complex have been reported for sheep and goats but not for cattle. Goats seem to be at a higher risk of having a *C. burnetii* associated abortion than other ruminants. Studies on other domestic mammals consistently show that they may become infected and develop antibodies but the outcome for the conceptus remains to be elucidated.

A number of studies have evaluated the association between infection with *C. burnetii* and a range of reproductive disorders other than abortion, especially in cattle. However, there is no solid evidence to support a hypothesis of *C. burnetii* causing disorders such as subfertility, endometritis/metritis, or retained fetal membranes. An association between *C. burnetii* and subclinical mastitis in dairy cattle may exist. This issue has not been investigated for other animal species. Epidemiological studies using appropriate controls should be done before treatment or prevention of such disorders is directed against *C. burnetii*.

## Abbreviations

APSW Complex: Abortion, Premature delivery, Stillbirth and Weak offspring Complex; *C. burnetii*: *Coxiella burnetii*; ELISA: Enzyme-linked immunosorbent assay; FISH: Fluorescent in situ hybridisation; IHC: Immunohistochemistry; Iv: Intravenously; *N. caninum*: *Neospora caninum*; PCR: Polymerase chain reaction; Q fever: Query fever; Sc: Subcutaneously.

## Competing interests

I have been invited by Ceva Animal Health Denmark, a manufacturer of a vaccine against Q fever, to participate in a meeting on Q fever (2011) and received salary for giving presentations (reviews) on Q fever for Danish veterinary practitioners (2011). Ceva Animal Health Denmark has financially supported one of my veterinary master students, who studied the seroprevalence of Q fever in Danish horses. This review was written independently by me and without regard to commercial interests.

I am an editor of Acta Veterinaria Scandinavia and from January 1, 2013 Editor-in-Chief. I have not been involved in the handling of my submission and have not in any way interacted with the review process or editorial decision making. A free waiver was granted by the journal for this manuscript.

## Authors’ information

I have been involved in research and diagnostic on reproductive disorders in domestic mammals since 1989. I did a PhD in veterinary pathology (1989–1991) focusing on bovine perinatal pathology at the Royal Veterinary and Agricultural University (now a part of the University of Copenhagen), Denmark. I was employed 1992–2000 at the Danish Veterinary Institute as a researcher/senior researcher and diagnostic pathologist with reproductive pathology of production animals as my key research area. This was followed by employment as associate professor in veterinary pathology (2000–2009) and professor in veterinary reproduction and obstetrics (2009 - ) at the University of Copenhagen, Denmark. I have been project manager and active researcher in a study on Q fever in Danish cattle.

## Supplementary Material

Additional file 1 Search strategy and selection criteria for references. Click here for file
