# Supplementary material for: Coxiella burnetii associated reproductive disorders in domestic animals-a critical review
Source: Acta Vet Scand. 2013 Feb 18;55(1):13. doi: 10.1186/1751-0147-55-13 (PMC3577508; doi:10.1186/1751-0147-55-13)
Supplement: Additional file 1 — Search strategy and selection criteria for references. [file 1751-0147-55-13-S1.docx]

# Search strategy and selection criteria

References for this Review were identified by searching PubMed [<http://preview.ncbi.nlm.nih.gov/pubmed/>] on September 17^th^ 2012 and again on November 11^th^ 2012 by the use of the terms “*Coxiella burnetii*” and “Q fever” in combination with each of the following species names: cattle, sheep, goat, buffalo, horse, pig, dog and cat. Articles resulting from these searches were considered. Selection was based on the title and in case of doubt, the abstract was consulted. Articles published in English, German, Danish, Swedish or Norwegian were included. Full-text articles dealing with the association between *C. burnetii*/Q fever and any aspects of reproduction in the domestic mammals mentioned were obtained either electronically or in printed version. In addition, relevant references cited in the articles fulfilling the search criteria were included in the Review if considered peer-reviewed. Articles were also identified through searches of the author’s own files and references cited in veterinary textbooks dealing with *C. burnetii*/Q fever and ruminant reproduction. Abstracts, including abstracts of publications in other languages than stated previously, articles or abstracts in proceedings, personal observations and unpublished data, empiric indications, web site information, reports and similar information not considered peer-reviewed were not included unless being central to the topic and assessed to be of sufficient reliability, e.g. the publication should contain sufficient details on materials and methods to allow independent evaluation of the results.

The review is based on the original publications when dealing with reproduction, while only a few selected publications are referred to when dealing with other issues such as excretion in milk, seroprevalence in general, zoonotic implications and vaccine efficacy.
